# Assessing the Biocontrol Activity of *Debaryomyces hansenii* Against Spoilage Molds in Synthetic and Meat-Derived Media

**DOI:** 10.3390/jof11090681

**Published:** 2025-09-19

**Authors:** Francisco J. Ruiz-Castilla, Ana L. Pérez-Fernández, Pablo I. Villamagua-Rojas, Helena Chacón-Navarrete, José Ramos

**Affiliations:** Department of Agricultural Chemistry, Edaphology and Microbiology, University of Córdoba, 14071 Cordoba, Spainb62chnah@uco.es (H.C.-N.)

**Keywords:** *Debaryomyces hansenii*, biocontrol, meat-based media, spoilage molds, fungal inhibition

## Abstract

The increasing interest in natural preservatives has driven the search for effective microbial agents capable of controlling spoilage molds in cured meat products. In this study, the efficacy of *Debaryomyces hansenii* strains as biocontrol agents against spoilage molds in dry-cured meat products was evaluated through a dual experimental approach using both synthetic (PDA) and meat-derived media (LBM). While all *D. hansenii* strains demonstrated strong antifungal activity in nutrient-rich synthetic media, their performance in meat-like conditions was moderate to high, with significant differences depending on the mold species, the yeast strain, and their interaction with the culture medium. Our results highlight that antifungal efficacy is strongly influenced by the growth environment, underscoring the limitations of traditional in vitro assays that depend solely on synthetic media. Incorporating food-mimicking systems early in the screening process proved critical to identify strain–mold–medium combinations with the highest potential. These findings support the potential application of native *D. hansenii* strains as natural preservatives to enhance the safety and shelf life of dry-cured meats, emphasizing the importance of testing in conditions that closely resemble the target food environment to select the most effective biocontrol solutions.

## 1. Introduction

In recent years, the growing consumer demand for safer and healthier food products has driven the industry to reduce or replace synthetic additives with natural alternatives. One of the primary consequences of this transition is the increased vulnerability of food matrices to microbial spoilage, particularly by filamentous fungi capable of altering the characteristics of the product, resulting in a reduction in its quality in terms of food safety and organoleptic qualities. Among the strategies explored to address this issue, the use of biocontrol agents has gained relevance due to their ability to act synergistically with existing preservation technologies while maintaining the overall quality of the food product [1,2,3,4,5].

Yeasts have emerged as promising candidates in this context due to their versatility, resilience, and ability to antagonize spoilage fungi through multiple mechanisms, including competition for nutrients and space, secretion of antifungal metabolites, biofilm formation, and production of volatile organic compounds (VOCs) [6,7,8]. Within this group, *Debaryomyces hansenii* stands out as a non-conventional yeast frequently isolated from salted and fermented foods, particularly dry-cured meat products and cheeses [1,4,9,10,11]. Its tolerance to high salt, low pH, and oxidative stress, along with its contribution to the sensory development of food products, makes it an excellent candidate for inclusion in integrated biopreservation systems [12,13,14,15,16,17].

*D. hansenii* belongs to the CTG clade of hemiascomycetous yeasts and exhibits genomic and physiological traits that clearly distinguish it from other model organisms such as *Saccharomyces cerevisiae* [18,19]. It shows remarkable genetic variability among strains, including differences in chromosome number and gene content [20,21], which may account for its strain-dependent efficacy as a biocontrol agent [4,22]. Moreover, the European Food Safety Authority (EFSA) includes *D. hansenii* in its Qualified Presumption of Safety (QPS) list, supporting its safe use in food fermentation and preservation processes [23]. Several studies have demonstrated the effectiveness of *D. hansenii* in inhibiting common spoilage fungi in food, including species of *Penicillium*, *Aspergillus*, *Mucor*, and *Fusarium*, through diverse antagonistic strategies [4,6,22,,24,25]. In dry-cured meat products, some of these molds pose a particular risk, as they can cause sensory defects or accumulate undesirable metabolites, especially under conditions with reduced preservative content that may favor fungal proliferation [26].

*D. hansenii* exerts its antifungal activity through a complex combination of mechanisms, the relative contribution of which has not yet been fully elucidated. Although this yeast is widely recognized as having a multifactorial nature in its antagonistic potential, most available studies have focused only on isolated aspects of its mode of action. Commonly described strategies include biofilm formation on food surfaces, which acts as a physical barrier against fungal colonization; competition for essential nutrients and ecological space, thereby limiting resource availability for spoilage fungi [6,7,22,25]; and the potential production of toxic compounds, such as killer proteins or other molecules with direct antifungal effects [27,28,29,30]. However, a deeper and integrated investigation of these mechanisms under controlled conditions and within real food matrices is still necessary to determine the key factors driving its efficacy more accurately as a biocontrol agent. Furthermore, the food matrix itself can strongly influence the expression of antifungal properties by *D. hansenii*. While synthetic media allow higher reproducibility in in vitro assays, they do not faithfully reproduce the physicochemical complexity of real products. When *D. hansenii* is cultivated in media prepared with cured meat, significant differences have been observed in both the volatile compound profile and the degree of fungal inhibition [31]. These findings underscore the need to design in vitro experimental systems that realistically simulate the target food product.

In the present study, a comparison of the antifungal potential of several *D. hansenii* strains isolated from cured Iberian pork loin was performed, focusing on their ability to inhibit representative spoilage molds under in vitro conditions. Two experimental systems were employed: PDA (potato dextrose agar) plates and a semi-synthetic medium prepared with homogenized Iberian pork loin and water. This dual approach allowed us to investigate whether the composition of the medium influences the antagonistic activity of *D. hansenii* against different spoilage molds associated with dry-cured Iberian pork loin to identify the most promising biocontrol candidates. The results obtained portrayed the relevance of early inclusion on food-based media when working in application studies to the food industry.

## 2. Materials and Methods

### 2.1. Biological Material and Culture Conditions

*D. hansenii* and mold strains used in this study were previously isolated from traditionally dry-cured Iberian pork loin (“*Lomo ibérico curado*”) produced in the Los Pedroches region (Córdoba, Spain) and maintained in the strain collection of the Department of Agricultural Chemistry, Edaphology and Microbiology at the University of Córdoba. The laboratory wild-type strain CBS767 (Fungal Biodiversity Center, The Netherlands) was also used as control (Table 1).

For experimental assays, all yeast and mold strains were grown on yeast extract peptone dextrose agar (YPDA; 1% yeast extract, 2% peptone, 2% D-glucose, and 2% agar, resulting in a final pH of 6.4 ± 0.2) or potato dextrose agar (PDA; 39 g/L OXOID, pH 5.4–5.8, Basingstoke, Hampshire, UK) and incubated at 26 °C. To simulate food-like conditions, a custom semi-synthetic meat-derived (Iberian cured pork loin) medium (LBM) was prepared by homogenizing cured Iberian pork loin in sterile distilled water (20% *w*/*v*) and 2% agar, resulting in a final pH of 6.0 ± 0.2. The homogenization was performed using a food processor until obtaining a uniform paste, and the homogenate was directly mixed with agar to prepare the LBM medium.

### 2.2. Evaluation of the Antifungal Potential of D. hansenii Against Undesirable Molds

The antagonistic activity of *D. hansenii* strains was assessed under in vitro conditions using two solid media: (i) standard PDA and (ii) LBM autoclaved at 121 °C for 20 min. Yeast suspensions were adjusted to 10^6^ CFU/mL by Neubauer chamber cell counting and 100 µL was evenly spread onto the surface of each plate. After, 10 µL of mold spore suspension (10^4^ spores/mL) was inoculated at the center of each plate. Plates were incubated at 26 °C for 10 days, and mold growth was monitored daily. All experiments were repeated at least three times, and three technical replicates for each sample were performed.

### 2.3. Data Quantification and Statistical Analysis

Inhibitory activity was quantified by measuring the radial expansion of the mold colony using ImageJ software (v1.53). In cases where molds grew as multiple or irregular colonies, the total mycelial area was measured instead, and the inhibition percentage was calculated based on these area measurements rather than a single diameter value. The inhibition percentage (IA) was calculated using the following formula:

IA (%) = [(C − T)/C] × 100

where C corresponds to the average diameter of the mold colony in the absence of yeast (control), and T is the diameter observed in co-cultivation with *D. hansenii*.

To determine whether the inhibition achieved by each *D. hansenii* strain differed significantly from that of the reference strain within each mold species, a Dunnett’s test was performed using GraphPad Prism v9 (Dotmatics, Boston, MA, USA). Graphs were prepared in Microsoft Excel, and the corresponding significance levels were added to each plot. Statistically significant differences are indicated as follows: *p* < 0.05 (*), *p* < 0.01 (**), and *p* < 0.001 (***).

To evaluate whether the antifungal activity of *D. hansenii* was influenced by the culture medium, yeast strain, and mold species, a three-way analysis of variance (ANOVA) was performed, with medium (PDA vs. LBM), strain (CBS767, LR2, SRF1, CB1), and mold species (*A. flavus*, *P. echinulatum*, *P. rubens*, *P. crustosum*) as fixed factors. Interaction terms (medium × strain, medium × mold, and medium × strain × mold) were included to determine whether the effect of the medium varied depending on strain and/or mold species. When significant effects were detected, Šidák’s multiple comparisons test was applied to identify differences between specific factor combinations. Assumptions of normality and homogeneity of variance were assessed using Q–Q plots of residuals and Levene’s test, respectively. These analyses were carried out using JASP (v0.95; University of Amsterdam, Amsterdam, The Netherlands).

## 3. Results

### 3.1. Inhibitory Potential of Debaryomyces hansenii Against Spoilage Molds on PDA Media

**Figure 1:**
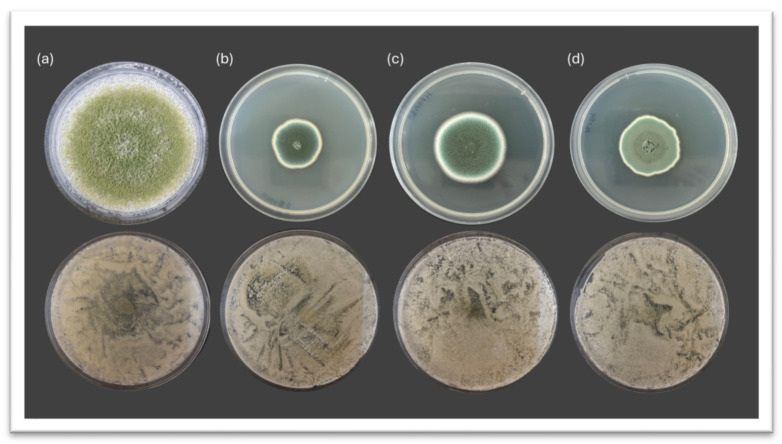
Representative images of the antifungal activity of different *Debaryomyces hansenii* strains against mold growth on PDA medium. Control plates without yeast (**top**) and yeast-treated plates (**bottom**) after 10 days of incubation at 26 °C. (**a**) *A. flavus*, (**b**) *P. echinulatum*, (**c**) *P. rubens*, and (**d**) *P. crustosum*.

After 10 days of incubation at 26 °C, strong antifungal activity was observed across all tested *D. hansenii* strains (CBS767, CB1, LR2, SRF1) against the four mold species evaluated on PDA plates: *A. flavus*, *P. echinulatum*, *P. rubens*, and *P. crustosum*. Macroscopic inspection revealed an evident reduction in mold growth in yeast-treated conditions compared to the untreated controls (Figure 1).

In the case of *A. flavus* (Figure 1a), the control plate exhibited dense and expansive mycelial growth, completely covering the surface, whereas the co-inoculated condition showed severely restricted mold development. Although not as visually impactful, the diminish in mycelial expansion was also clear for *P. echinulatum* (Figure 1b), *P. rubens* (Figure 1c), and *P. crustosum* (Figure 1d), where yeast-treated plates exhibited fragmented, irregular, and diminished mycelial patterns compared to the controls. These visual results support the potent antagonistic activity of *D. hansenii* against spoilage mold on rich nutrient laboratory media.

**Figure 2:**
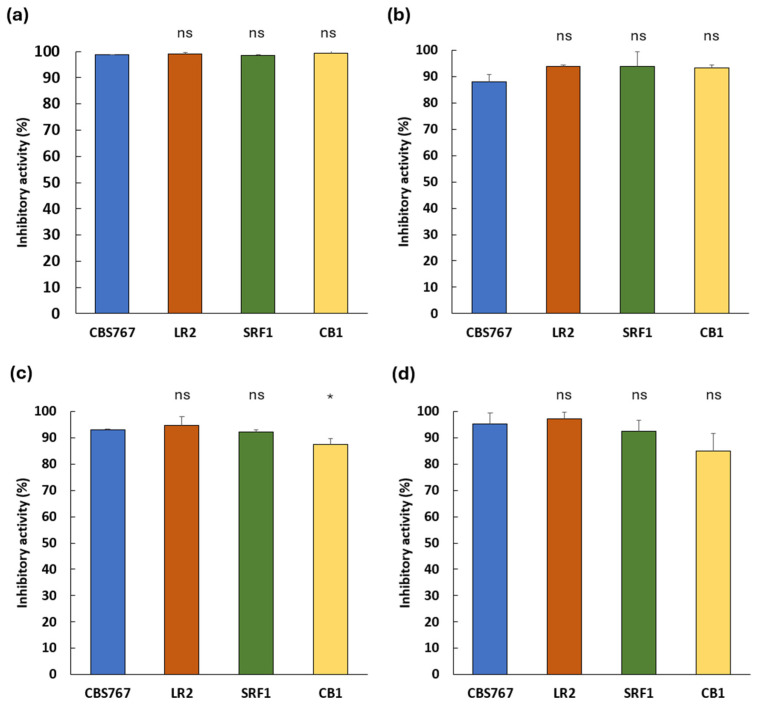
Inhibition percentage of each *D. hansenii* strain against mold contaminants on PDA media. (**a**) *A. flavus*, (**b**) *P. echinulatum*, (**c**) *P. rubens*, (**d**) *P. crustosum*. Bars represent means ± SD (n ≥ 3). In most cases, ns: no significant differences were observed (ANOVA; *p* > 0.05), *p* < 0.05 (*).

Quantitative assessment of the antifungal activity is shown in Figure 2, where inhibition percentages for each *D. hansenii* strain are presented across the four mold species.

The highest inhibition was observed against *A. flavus* (Figure 2a), with all yeast strains reaching inhibition percentages above 98%. For the remaining mold species (*P. echinulatum*, *P. rubens*, and *P. crustosum*; Figure 2b–d), inhibition values ranged from 85% to 97%, indicating a consistently high antifungal performance across all yeast strains.

Notably, strain LR2 achieved the greatest inhibitory activity overall, with inhibition levels exceeding 93% against *P. echinulatum* (93.77%), *P. rubens* (94.57%), and *P. crustosum* (97.10%). In most cases, no statistically significant differences were observed between the native isolates and the reference strain CBS767. For CB1 strain against *P. rubens*, which, although low, showcased significantly lower inhibition from a 93% in CBS767 to 88% in CB1. Altogether, these results underscore the robust and uniform antifungal potential of *D. hansenii* under standard rich nutrient media and laboratory conditions.

### 3.2. Inhibitory Potential of Debaryomyces hansenii Against Spoilage Molds on Meat-Derived Media

**Figure 3:**
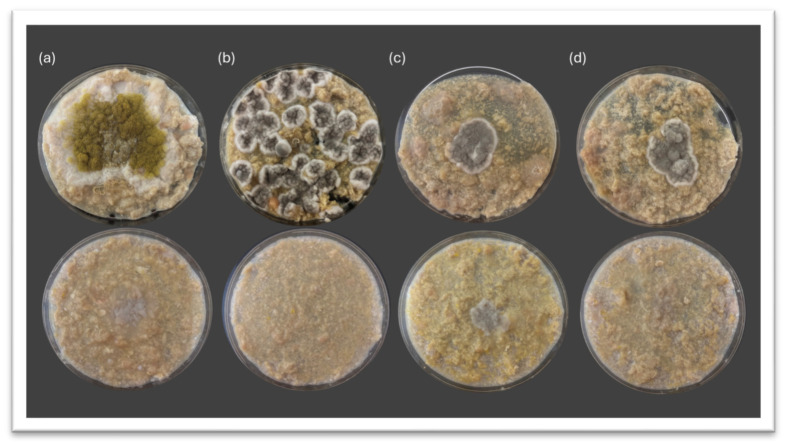
Representative plates showing mold growth on LBM with and without yeast treatment. Control plates (**top**) and yeast-treated plates (**bottom**) after 10 days of incubation at 26 °C. (**a**) *A. flavus*, (**b**) *P. echinulatum*, (**c**) *P. rubens*, and (**d**) *P. crustosum*.

To better simulate the cured meat environment, the antagonistic activity of *D. hansenii* was further assessed on LBM. Although the overall inhibition percentages were lower than those determined in PDA, clear antifungal activity was still detected (Figure 3).

In the control plates (top row of each panel), fungi exhibited vigorous radial expansion typical of their growth under nutrient-rich conditions. In contrast, the yeast-treated plates (bottom row) showed visibly reduced mycelial development across all tested fungi. The most pronounced inhibition was observed in panel (Figure 3b,d), where *P. echinolatum* and *P. crustosum* growth was visibly diminished. In panel (Figure 3a), *A. flavus* still showed moderate development, suggesting that its suppression may be more influenced by medium composition. These results support that *D. hansenii* maintains its antagonistic activity in meat-like substrates, although matrix complexity and nutrient availability likely modulate the extent of inhibition.

**Figure 4:**
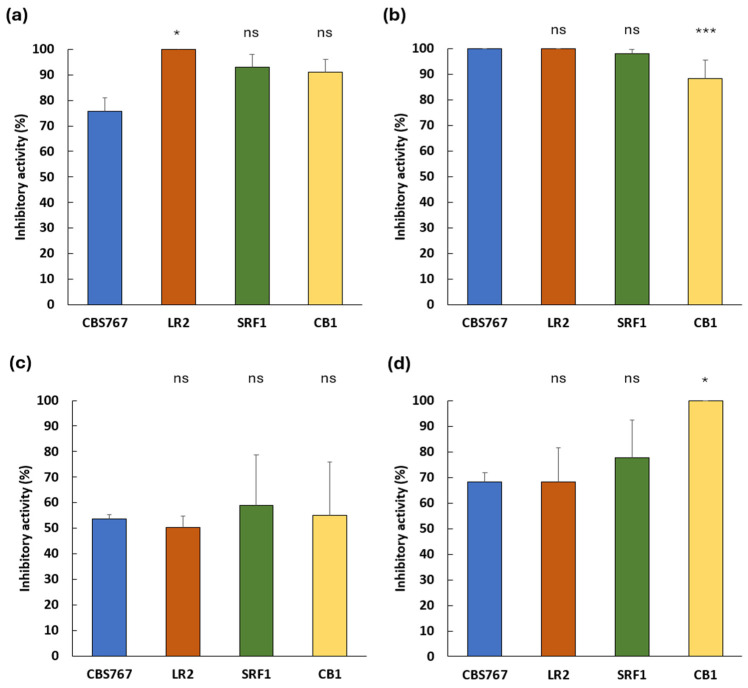
Inhibition percentage of each *D. hansenii* strain against mold contaminants on LBM. Bars represent means ± SD (n ≥ 3). (**a**) *A. flavus*, (**b**) *P. echinulatum*, (**c**) *P. rubens*, (**d**) *P. crustosum*. Bars represent means ± SD (n ≥ 3). In most cases, ns: no significant differences were observed (ANOVA; *p* > 0.05), *p* < 0.05 (*), *p* < 0.001 (***).

Quantitative assessment of the antifungal activity is shown in Figure 4, where inhibition percentages for each *D. hansenii* strain are presented across the four mold species.

Overall, antifungal activity was maintained under these more complex matrix conditions, although the magnitude of inhibition varied depending on the mold species and yeast strain. Inhibition percentages ranged from 50% to 100%, with greater variability observed compared to PDA assays.

For *A. flavus* (Figure 4a), all strains achieved inhibition levels above 70%, with LR2 strain exhibiting significantly higher activity (100%) than the reference strain CBS767 (*p* < 0.05). In contrast, most strains inhibited *P. echinulatum* (Figure 4b) uniformly and strongly (>95%), with a lower inhibition (88%, *p* < 0.001) observed in strain CB1. Inhibition of *P. rubens* (Figure 4c) was lower and more heterogeneous, ranging from ~52% to ~64%, with SRF1 displaying the most consistent and robust inhibition, though differences were not statistically significant. For *P. crustosum* (Figure 4d), inhibition ranged from ~70 to 96%, with CB1 showing the highest mean value (100%, *p* < 0.05). These results confirmed that *D. hansenii* retains antifungal activity under meat-like conditions. However, despite maintaining its inhibitory capacity, the efficacy varied depending on the culture media used, highlighting the influence of the substrate composition on the biocontrol performance.

### 3.3. Influence of the Culture Medium on the Antifungal Performance of Debaryomyces hansenii

The three-way ANOVA, with medium (PDA vs. LBM), strain (CBS767, LR2, SRF1, CB1), and mold species (*A. flavus*, *P. echinulatum*, *P. rubens*, *P. crustosum*) as fixed factors, revealed that the main effect of medium was significant (*p* < 0.001), indicating that, overall, the type of culture medium influenced the antifungal activity of *D. hansenii* (Table 2). Yeast strain did not have a significant main effect (*p* = 0.204), whereas mold species showed a strong effect (*p* < 0.001), confirming that the target fungus markedly affected inhibition levels.

Among the interactions, Medium × Yeast strain (*p* = 0.031), Medium × Mold (*p* < 0.001) and Medium × Yeast strain × Mold (*p* = 0.001) were significant, while Yeast strain × Mold (*p* = 0.179) were not. This pattern indicates that the effect of the medium was not uniform across all conditions: the magnitude and direction of the medium effect varied depending on the mold species, the strain of *D. hansenii*, and, in some cases, their specific combination.

Post hoc Šidák’s tests confirmed that inhibition was significantly higher on PDA than on LBM for *P. rubens* (*p* < 0.001) and *P. crustosum* (*p* = 0.048), whereas no significant differences between media were observed for *A. flavus* and *P. echinulatum*. The magnitude of these differences was also strain-dependent in certain mold species. For instance, LR2 *D. hansenii* strain showed the largest drop in inhibition against *P. rubens* when moving from PDA to LBM, whereas CBS767 maintained similar inhibition levels against *A. flavus* across both media.

## 4. Discussion

*D. hansenii* is a yeast with remarkable physiological versatility [19,32] and a multifactorial mode of action as a biocontrol agent [6,7]. Its antagonistic capacity against molds is not limited to a single mechanism but rather involves multiple strategies, including competition for nutrients and space, biofilm formation, the production of VOCs, and the synthesis of specific mycocins [22,23,,24,25]. This diversity of mechanisms may act synergistically or differentially depending on environmental conditions, making it reasonable to assume that, in complex matrices such as those derived from meat, certain mechanisms may lose prominence while others may become more relevant.

As all scientific studies whose goal is to bring novelty to industry, laboratory-scale assays are needed before scaling up processes. Frequently, this type of study, although promising, ends up not being suitable for a bigger scale, turning this into a waste of resources and time. Laboratory assay reliability depends on the ability to simulate industrial conditions and on the use of appropriate validation and monitoring tools. Predictions are more accurate when models and devices specifically designed to reflect the complexity of industrial processes are employed [33,34,35]. *D. hansenii* biocontrol potential-related studies usually employ standardized laboratory culture media that allow results to be compared and mechanisms of inhibition to be understood. One of the most widely used synthetic media in fungi-related studies is PDA [36,37,38,39,40,41] being also frequently used medium in *D. hansenii* biocontrol assays [4,6,17,22].

The results of this study, supported by the three-way ANOVA presented in Table 2, show that the culture medium had a significant overall effect on the antifungal efficacy of *D. hansenii* (*p* < 0.001), and that the medium × yeast strain interaction was also significant (*p* = 0.031), indicating that the effect of the medium varied across strains when averaged over all molds. The main effect of yeast strain was not significant (*p* = 0.204). Under synthetic conditions using PDA media, all strains of this study exhibited strong inhibitory activity against spoilage molds, with inhibition rates exceeding 85%, and particularly high performance by LR2 strain, which achieved over 93% inhibition. This behavior was consistent with previous studies reporting the ability of *D. hansenii* to inhibit the growth of toxigenic molds in nutrient-rich media such as PDA [4,6,17,22]. However, when simulating conditions closer to the actual food environment by using a medium composed of 20% meat (LBM), the antifungal efficacy was generally reduced. This confirms that the growth substrate is a primary driver of antifungal performance in this system, with potential implications for industrial-scale applications. The effect of the medium was not uniform across all molds, as indicated by the significant medium x mold interaction (*p* < 0.001), and in specific cases also varied depending on the strain–mold combination, as reflected in the significant medium × yeast strain × mold interaction (*p* = 0.001). Significant reductions in inhibition were observed for *P. rubens* and *P. crustosum* in the LBM medium, while inhibition against *A. flavus* and *P. echinulatum* remained comparable to PDA. Despite this, *D. hansenii*’s strains maintained moderate to high inhibition levels, reinforcing their applied potential. These findings confirm that biocontrol performance in PDA does not necessarily predict performance in a meat-based medium, and highlight that the relative ranking of strains can change depending on the growth substrate, target mold and their interaction with the yeast strain tested. This variability supports the inclusion of food-derived or matrix-mimicking media in early screening stages, as results from synthetic systems may overestimate the true antagonistic potential of candidate strains.

Despite the general reduction in inhibitory activity observed in the meat-derived media compared to the synthetic medium (PDA), the inhibition levels remain relevant in an applied context. The maintenance of inhibitory activity above 50% against spoilage molds in a complex matrix such as cured meat suggests that these yeasts hold real potential as protective cultures. This discrepancy between synthetic and food-like media in *D. hansenii* studies is not unique to our findings. García-Bramasco et al. [42] demonstrated that *D. hansenii* embedded in chitosan-based films showed strong antifungal activity against *Penicillium italicum*, but the effectiveness varied with matrix composition. This observation echoes findings by Huang et al. [9], who noted that VOC-mediated inhibition by *D. hansenii* strains varied significantly across dairy matrices. Consequently, this supports the idea, already observed in earlier studies [31,43,44], that functional assays using food-simulating matrices should be an essential part of the selection and validation process for promising protective cultures prior its application in the industry.

As repeatedly reported in the literature, one of the main struggles in the study of *D. hansenii* and its applications is its high variability among strains [20,21]. This study underscores that when applying meat-like matrices, this variability becomes less marked, turning this matrix more effective and reliable to scale up to practical applications. Consequently, validating biocontrol performance in food-like systems emerges as a superior strategy, ensuring a more precise selection of strains that can genuinely contribute to enhancing food safety and shelf life in meat products.

Overall, this study demonstrates that *D. hansenii* has strong potential as a biocontrol agent against spoilage molds in dry-cured meat products. The dual-phase evaluation strategy employed in this study, combining synthetic and meat-derived media, highlights the importance of assessing antifungal effectiveness under conditions that closely mimic the final product environment. All autochthonous strains evaluated, especially SRF1 and LR2, proved capable of maintaining substantial inhibitory activity even in a meat-derived media, reinforcing their value as candidates for future technological applications in the meat industry.

## 5. Conclusions

The findings of this study provide new evidence supporting the potential of *D. hansenii* as a biocontrol agent against spoilage molds in dry-cured meat products. All tested strains exhibited strong inhibitory activity in synthetic media, with strain LR2 showing the highest inhibition rates. Although a general decrease in efficacy was observed in the meat-derived medium, *D. hansenii*’s strains maintained considerable inhibitory capacity, highlighting their relevance for practical applications.

The significant effect of the culture medium, together with yeast strain and mold interactions, demonstrates that antifungal efficacy is strongly influenced by both the composition of the growth matrix and the specific strain–mold combination. While synthetic media remain valuable for initial screenings and mechanistic studies, they should not be the sole basis for selecting biocontrol agents. The integration of food-simulating matrices provides a more accurate representation of matrix-dependent and strain-specific interactions and enhances the reliability of strain selection. Our findings emphasize the importance of including food-mimicking systems in the early stages of biocontrol screening to better predict performance in real food environments. Accordingly, we recommend incorporating representative food-based media in early phases of biocontrol strategy development to ensure more realistic and effective strain selection.

Altogether, this work identifies promising native *D. hansenii* strains with consistent biocontrol potential under conditions that closely resemble the dry-cured meat production process. Their application as protective cultures could contribute to improving the safety and shelf life of these traditional products, offering a more natural and consumer-friendly alternative to chemical preservatives.

## Figures and Tables

**Table 1:** Microorganisms used in this study.

Microorganism	Strain	Reference
*D. hansenii*	CBS767 (Wild type reference)	[12]
*D. hansenii*	LR2	[10]
*D. hansenii*	SRF1	[10]
*D. hansenii*	CB1	[10]
*Aspergillus flavus*	HSC31	[22]
*Penicillium echinulatum*	HPDA3	[22]
*Penicillium rubens*	HPDA10	[22]
*Penicillium crustosum*	HSC30	[22]

**Table 2:** Summary of the three-way ANOVA evaluating the effects of medium, strain, and mold species, and their interactions, on the antifungal activity of *D. hansenii*.

Factor/Interaction	df	F-Value	*p*-Value
Medium	1	80.667	<0.001
Yeast strain	3	1.579	0.204
Mold	3	46.268	<0.001
Medium × yeast strain	3	3.165	0.031
Medium × mold	3	35.385	<0.001
Yeast strain × mold	9	1.473	0.179
Medium × yeast strain × mold	9	4.066	0.001

## Data Availability

The original contributions presented in this study are included in the article. Further inquiries can be directed to the corresponding author.
